# Normal and Functional TP53 in Genetically Stable Myxoid/Round Cell Liposarcoma

**DOI:** 10.1371/journal.pone.0113110

**Published:** 2014-11-13

**Authors:** Anders Ståhlberg, Christina Kåbjörn Gustafsson, Katarina Engtröm, Christer Thomsen, Soheila Dolatabadi, Emma Jonasson, Chieh-Yuan Li, David Ruff, Shiaw-Min Chen, Pierre Åman

**Affiliations:** 1 Sahlgrenska Cancer Center, Department of Pathology, Institute of Biomedicine, University of Gothenburg, Gothenburg, Sweden; 2 Department of Oncology, Institute of Medical Sciences, University of Gothenburg, Gothenburg, Sweden; 3 Genetic, Medical and Applied Sciences division, Life Science Group, Thermo Fisher Scientific, South San Francisco, CA, United States of America; Hertie Institute for Clinical Brain Research and German Center for Neurodegenerative Diseases, Germany

## Abstract

Myxoid/round-cell liposarcoma (MLS/RCLS) is characterized by either the fusion gene *FUS-DDIT3* or the less commonly occurring *EWSR1-DDIT3* and most cases carry few or no additional cytogenetic changes. There are conflicting reports concerning the status and role of TP53 in MLS/RCLS. Here we analysed four MLS/RCLS derived cell lines for *TP53* mutations, expression and function. Three SV40 transformed cell lines expressed normal TP53 proteins. Irradiation caused normal posttranslational modifications of TP53 and induced P21 expression in two of these cell lines. Transfection experiments showed that the FUS-DDIT3 fusion protein had no effects on irradiation induced TP53 responses. Ion Torrent AmpliSeq screening, using the Cancer Hotspot panel, showed no dysfunctional or disease associated alleles/mutations. In conclusion, our results suggest that most MLS/RCLS cases carry functional TP53 genes and this is consistent with the low numbers of secondary mutations observed in this tumor entity.

## Introduction

A majority of common human sarcoma entities carry complex and heterogeneous chromosome aberrations typical for tumors with genomic instability. A smaller group contain few chromosome aberrations and are typically characterized by simple recurrent chromosome rearrangements that result in formation of tumor type specific fusion oncogenes [1]. Most sarcomas carrying FET (*FUS*, *EWSR1*, *TAF15*, also known as TET) family fusion oncogenes have few or no cytogenetic changes except for the rearrangements that generate the fusion oncogenes [2]. Myxoid/round cell liposarcoma (MLS/RCLS) characterized by either the t(12;16) or the t(12;22) translocation, is one of the most common FET oncogene carrying sarcomas. The specific fusion oncogenes consist of the 5′partners *FUS* (also known as *TLS*) or more rarely *EWSR1*, fused to the transcription factor gene *DDIT3* (also known as *CHOP* or *GADD153*) [3], [4]. The chimeric *FUS-DDIT3* and *EWSR1-DDIT3* encoded proteins are believed to function as abnormal DNA binding transcription factors that interfere with differentiation and growth control [5]–[8]. The importance of this function is further supported by the effect of Trabectedin treatment leading to detachment of FUS-DDIT3 from specific DNA binding sites [9]–[11]. More than 30% of the cases carry the translocation as the only cytogenetic aberration at diagnosis [12]–[14]. Besides of the fusion oncogene, mutations in *PIK3CA* or loss of *PTEN* expression is seen in 10–15% of the cases and these changes are associated with poor prognosis [12]. *TP53* mutations have also been reported and associated with progressive disease [13], [15]–[17]. The vast majority of the tumors carry normal *TP53* genes and secondary changes are few and rare even when relapses occur [14]. In contrast to genetically complex sarcomas, MLS/RCLS is highly sensitive to irradiation and chemotherapy [18], supporting the view that the tumor cells maintain a functional and responding TP53 system. A recent study shows however, that a *FUS-DDIT3* transgene fails to induce tumors in mice if not introduced into a *TP53* deficient genetic background [19]. This indicates that impaired TP53 function could be of importance in MLS/RCLS development.

In the present investigation we examined the TP53 protein in four MLS/RCLS derived cell lines, three with normal and one with a known mutated *TP53* gene. Functional TP53 analysis was performed using irradiation experiments with downstream western blot and immunofluorescence analyses. We also screened three MLS/RCLS derived cell lines for commonly occurring mutations using Ion Torrent AmpliSeq Cancer Hotspot Panel.

## Materials and Methods

### Cell culture

Cells and cell line characteristics are shown in Table 1. All cell lines were published before and cultured in RPMI1640 GlutaMAX medium supplemented with 10% fetal bovine serum, 100 U/mL penicillin and 100 µg/mL streptomycin at 37°C in 5% CO_2_. The FUS-DDIT3-EGFP transfected HT1080 cells [5] were cultured with 500 µg/ml of Geneticin until 24 hours before experiments. All media and supplements were obtained from Life Technologies. DL 221 cultured tumor cells, explanted from tumor tissues of an MLS/RCLS patient, were provided by Dr. Alexander Lazar. Acquisition of the tissue specimen was approved by the institutional review board of The University of Texas MD Anderson Cancer Center (UTMDACC), and performed in accordance with the Health Insurance Portability and Accountability Act regulations.

**Table 1:** Cell line characteristics.

Cell line	SV40 status	FUS-DDIT3 protein	Reference
MLS 402-91	Large T antigen	Type I fusion (*FUS* exons 1–7)	[3]
MLS 2645-94	Full SV40 virus	Type II fusion (*FUS* exons 1–5)	[24]
MLS 1765-92	Large T antigen	Type VI fusion (*FUS* exons 1–13)	[3]
DL 221 cells	Negative	Type I fusion (*FUS* exons 1–7)	Dr. Lazar
HT1080 EGFP	Negative	Negative	[5]
HT1080 FUS-DDIT3-EGFP	Negative	Type II fusion (*FUS* exons 1–5)	[5]

### Protein extraction, SDS-PAGE and western blot

Cells were washed with ice-cold Phosphate-Buffered Saline (PBS, Life Technologies) and collected in RIPA buffer (25 mM Tris-HCl, pH 7.6, 150 mM NaCl, 1% IGEPAL, 1% sodium deoxycholate, 0.1% SDS) (Pierce); supplemented with 1X Halt Protease and Phosphatase Inhibitor Cocktail (Thermo Scientific). Samples were incubated on ice for 10 min and cleared by centrifugation at 14000 g for 10 min at 4°C. The protein concentrations of samples were determined with the *DC* Protein Assay (BioRad) according to the manufacturer's recommendations. Samples were stored at −20°C.

SDS-PAGE and immunoblotting was performed with the Novex NuPAGE system (Life Technologies) according to the manufacturer's recommendations. The protocol is described in detail elsewhere [20]. The following primary antibodies were used: anti TP53 central parts (Pab240, diluted 1∶200, Santa Cruz Biotechnology), anti TP53 N-terminal (DO-1, diluted 1∶200, Calbiochem Merck), anti TP53 C-terminal (Pab421, diluted 1∶200, Calbiochem Merck), anti DDIT3 (15204-1-AP, diluted 1∶266, Proteintech) and anti GAPDH (mAbcam 9484, diluted 1∶200, Abcam)

### Immunohistochemistry

Paraffin embedded sections from seven MLS/RCLS cases and one endometrial carcinoma were obtained from our pathology department in conformity with Swedish legislation (approved by the Ethical Board associated with the University of Gothenburg). Immunohistochemistry was performed as described previously [5] using the TP53 specific antibody OP43A (Calbiochem Merck) at a dilution of 1∶100.

The histological specimens were examined and evaluated in a blinded fashion by two examiners. Stained tumor cells were counted at 200× magnification. Cells with nuclear and cytoplasmic expression were counted avoiding inflammatory cells, endothelial cells and necrotic areas. Three different areas in each slide were counted and a mean value was calculated. To assess weakly or strongly stained cells 500 cells in five different areas or more were analyzed.

### Immune precipitation and mass spectrometry

Cells where washed once with PBS and collected in ice-cold lysis buffer (25 mM Tris pH 7.5, 1% CHAPS, 100 mM NaCl) supplemented with HALT Protease and Phosphatase Inhibitor Cocktail (Pierce). The cell suspension was cleared by centrifugation at 1000 rcf for 10 min at 4°C. Protein extracted from approximately 10^7^ cells was incubated with 5 µg of anti TP53 antibody (Pab1620, Abcam) in 1100 µl lysis buffer at 4°C for 1 h under gentle shake. Forty µl of pre-equilibrated DynaBeads Protein-A (Invitrogen) was added and the mixture was incubated for 1 h followed by washing in DynaMag-Spin (Invitrogen) with 750 µl lysis buffer (twice) and with 750 µl dilution buffer diluted 1∶2 (once). Captured proteins were eluted from the immobilized beads by adding 20 µl 0.2% acetic acid and incubating for 5 min at room temperature. Sample preparation and mass spectrometry analysis was performed at The Proteomics Core Facility at the Sahlgrenska Academy, University of Gothenburg as described [20].

### Immunofluorescence

Cells were fixed in 3.7% formaldehyde (Sigma-Aldrich) in PBS, pH 7.2 at 37°C, washed in PBS and stained against TP53 (FL-393, Santa Cruz Biotechnology) or P21 (EA10, Merck), both diluted 1∶100 in PBS supplied with 2% BSA (Sigma-Aldrich) and 0.2% Triton X-100 (Merck). Bound primary antibodies were detected using Cy3 secondary antibodies (PA43002 respective PA43004, Amersham). Slides were mounted using Prolong Gold anti-fade with DAPI (Invitrogen). Cellular fluorescence was imaged using a Zeiss Axioplan 2 fluorescence microscope (Zeiss).

### PCR and DNA sequence analysis

PCR fragments for *TP53* splice variants were amplified from cDNA of the cell lines and confirmed by sequencing as described [21]. The following PCR primers were used: TP53exon1_4 (forward: 5′-GGAGGAGCCGCAGTCAGAT-3′, reverse: 5′-TTCAATATCGTCCGGGGA-3′), TP53exon4_6 (forward: 5′-TCCCTTCCCAGAAAACCTACC-3′, reverse: 5′-ACCACACTATGTCGAAAAGTGTTTC-3′), TP53exon5_8 (forward: 5′-AAGCAGTCACAGCACATGAC-3′, reverse: 5-GCGGAGATTCTCTTCCTCTGT-3′), TP53exon8_10 (forward: 5′-TTTGTGCCTGTCCTGGGA-3′, reverse: 5′-TGGGCATCCTTGAGTTCC-3′) and TP53exon10_11 (forward: 5′-GAGCAGGGCTCACTCCAG-3′, reverse: 5′-TATGTCCTACTCCCCATCCTCC-3′)

Mutation screening was made using an Ion Torrent PGM sequencer with Ion 318 Chip and Life Technologies Ion AmpliSeq Cancer Hotspot Panel v2 (Life Technologies). The CHPv2.0 panel consists of 207 amplicons. The Ion AmpliSeq protocol for library preparation was used. The amplicon coordinates are documented at the vendor web site.

### Data Analysis – Ion PGM data

The DNA AmpliSeq Cancer Hotspot Panel v2 PGM data were aligned to the hg19 genome sequence and mapped to the CHPv2.0 bed file coordinates. The variants were called using the Torrent VariantCaller plugin in the Torrent Suite software. The variants were manually curated against the BAM file and only positive variants were reported.

## Results and Discussion

Most MLS/RCLS tumors are considered genetically stable and many cases carry the characteristic fusion gene associated translocation as the only chromosome aberration even when investigated in tumor recurrence after many years of remission. A limited number of MLS/RCLS derived established cell lines are available. Here, we investigated the TP53 function in four of these cell lines, all carrying the characteristic fusion oncogene *FUS-DDIT3* (Table 1).

Next generation sequencing and mutation analysis showed that MLS 402-91, 2645-94 and 1765-92 cell lines contained no dysfunctional or pathogenic alleles among the 50 genes, covering ∼2800 COSMIC mutations in the AmpliSeq Cancer Hotspot Panel (Table 2 and S1). This low mutation frequency is remarkable considering that these cell lines have a history with more than 20 years of passages. The DL 221 cells carry mutated *TP53* (personal communication with Dr. Alexander Lazar), which was confirmed by our sequence analysis (data not shown). Detailed sequence analysis of the DL 221 cells will be described elsewhere.

**Table 2:** Sequence variations in MLS cell lines.

					MLS 402-91^5^			MLS 2645-94^5^			MLS 1765-92^5^		
Gene	Chrlocation^1^	Codon change^2^	aa change^3^	Hot spot^4^	Zygosity	Genotype	Variant	Zygosity	Genotype	Variant	Zygosity	Genotype	Variant
*KDR*	4:55972974	CAA>CAT	Q ->H	COSM149673	–	–	–	Het	T/A	A			
*TP53*	17:7579472	CCC>CGC	P ->R	COSM45985	Het	G/C	C	Hom	C/C	C	Het	G/C	C

All variations are single nucleotide polymorphisms.

1 Genomic location at respective chromosome using hg19 genome sequence.

2 Codon change within the reading frame of respective gene.

3 Amino acid change due to the observed polymorphism. Both detected polymorphisms are normal and commonly occurring variants.

4 Cosmic annotated mutations with corresponding ID are indicated.

5 Variant observed. For example, for MLS1765-92 and *TP53* (Het, G/C, C) data indicate that the observed variation (“C”) is heterozygote, while the reference nucleotide is “G”. Note that the codon change for *TP53* is CCC>CGC, since the gene is located at the minus DNA strand. Het, heterozygote; Hom, homozygote.

**Figure 1:**
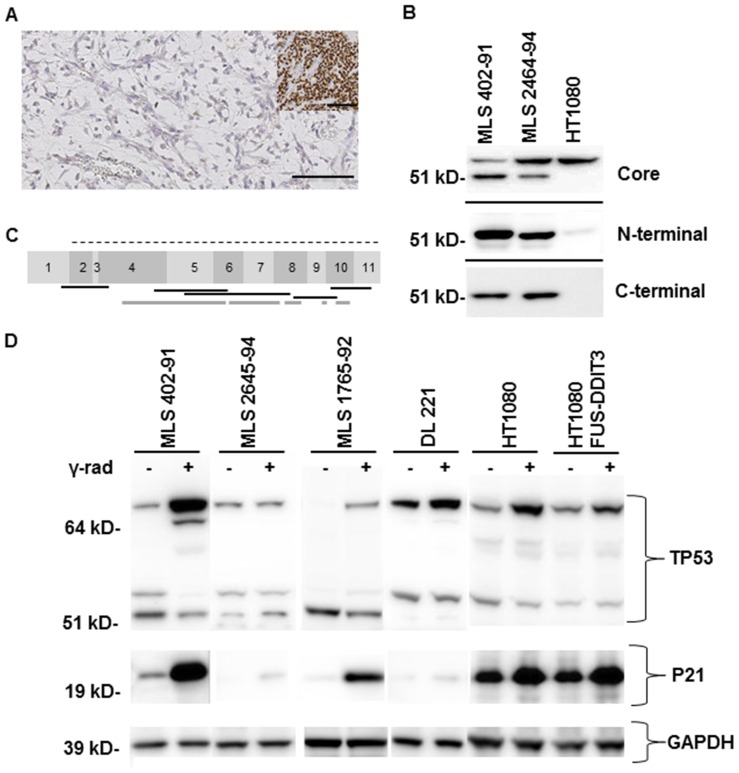
TP53 expression in MLS/RCLS. (A) Immunohistochemistry analysis of TP53 expression in a representative MLS/RCLS case. Inset shows TP53 mutated endometrial carcinoma as positive control. Brown precipitate indicates TP53 expression. Scale bar shows 100 µm. (B) Western blot analysis of TP53 in MLS and HT1080 cell lines. Three different antibodies directed against the core (central transcription factor part), N- and C-terminal parts were used. Two distinct bands (53 and 56 kD, respectively) were detected for the core part of TP53, while only the shorter 53 kD band was detected using antibodies against N- and C-terminal part of TP53. (C) Schematic map of eleven exons in *TP53*. The translated region is shown as dotted line, and only the translated part of exon eleven is shown. Transcripts detected by reverse transcription PCR and sequencing are shown as black lines. Protein fragment analyses by mass spectroscopy for MLS 402-91 are shown as grey lines. (D) Irradiation effect on TP53 and P21 (CDKN1A) expression in four MLS/RCLS cell lines, wild type HT1080 cells and HT1080 cells expressing FUS-DDIT3 (HT1080 FUS-DDIT3). Antibody against core TP53 part was used. The 68 KD band corresponds to post-translationally modified TP53. MLS 402-91 and 1765-92 carry simian virus 40 large T antigen, while MLS 2645-94 was established using the complete SV40 virus. GAPDH is used as loading control, +/− indicate irradiated and control cell samples, respectively. Positions and sizes of reference proteins are indicated.

TP53 is central in genomic maintenance and recent large scale sequencing of MLS/RCLS cases showed no *TP53* mutations among twenty-seven samples [12]. Mutations in *TP53* often results in overexpression of the mutated protein that can be visualized by immunohistochemistry analysis [22]. Hence, we analyzed seven cases and only sporadic *TP53* expressing cells were detected, suggesting normal TP53 function (Figure 1A).

### Elevated TP53 expression in MLS derived cell lines

**Figure 2:**
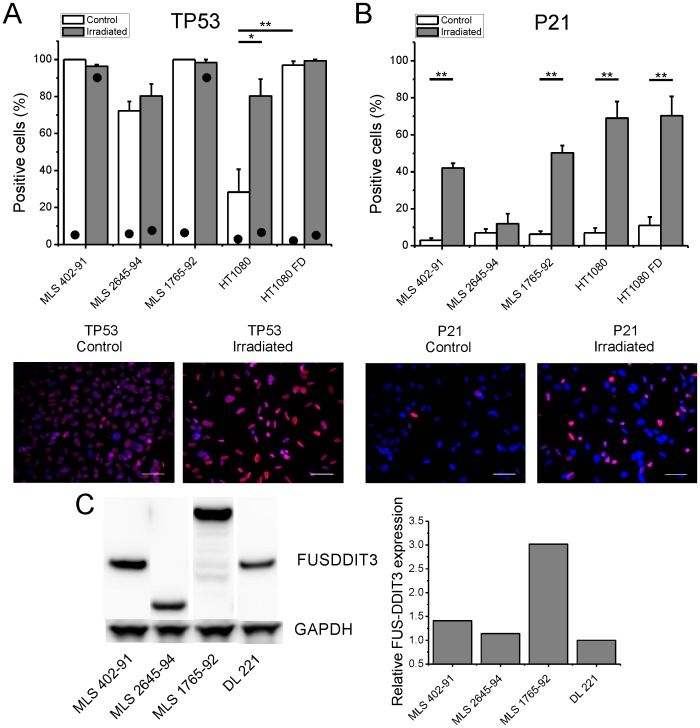
Immunofluorescence analysis of TP53 and P21 in irradiated and control cultured cells. Percentage of (A) TP53 and (B) P21 positive cells are shown. Mean ± SEM of three experiments are shown. * and ** indicate 95% and 99% significance using student's t-test. The number of strongly TP53 stained cells was assessed for one experiment, marked with dot (•) in respective bar. Representative immunofluorescence images for MLS 402-91 are shown. Scale bar shows 50 µm. (C) Western blot analysis of FUS-DDIT3 in MLS cell lines are shown. Different sizes correspond to Type I (MLS 402-91 and DL 221), II (MLS 2645-94) and VI (MLS 1765-92) fusion proteins. GAPDH is used as internal control between samples when calculating the relative FUS-DDIT3 expression level. The lowest FUS-DDIT3 expression value (DL 221) was arbitrarily set to a value of one.

In contrast to tumor tissues, the MLS/RCLS derived in vitro cultured cell lines MLS 402-91, 2645-94, 1765-92 and DL 221tumor cells all expressed TP53 protein as shown by our western blot (Figure 1B and 1D) and immune fluorescence analyzes (Figure 2). The reason for the elevated TP53 expression in cultured MLS/RCLS cells is not known. Cell stress caused by the *in vitro* culture conditions is one plausible explanation and is supported by the fact that freshly explanted cells from MLS/RCLS tissues rapidly start to express the TP53 protein, entering senescent stage within a few passages (data not shown). Transfection with SV40 large T antigen encoding vectors allowed for establishment of MLS cell lines, except DL221 [3]. The two MLS cell lines 402-91 and 1765-92 were stably transfected with expression vectors carrying simian virus 40 (SV40) large T antigen [3], [23]. MLS 2645-94 was established by infection with the complete SV40 virus (Table 1) [24]. The SV40 encoded proteins are reported to bind TP53 and interfere with its function [25], [26]. This is likely contributing to the survival and continued growth of the MLS cell lines in spite of high TP53 levels.

### Normal TP53 protein is produced in MLS derived cell lines

Western blot analysis showed a 53 kD band using three different antibodies directed against the TP53 N-terminal, core (DNA binding) and C-terminal domains in MLS 402-91 and 2645-94 (Figure 1B). A 56 kD band reacted with the TP53 core specific antibody, but not or very faintly with antibodies directed against the N- and C-terminal parts. HT1080 cells, reported to contain normal and functional TP53 alleles, expressed the larger 56 kD protein detected with the TP53 core antibody [27]. The N- and C-terminal specific TP53 antibodies showed weak reactivity in HT1080 (Figure 1B). MLS 1765-92 mainly expressed the 53 kB band, while DL 221 expressed the larger 56 kB band (Figure 1D).

The observation of several sized TP53 proteins prompted further investigation as TP53 isoforms may be produced from alternative splicing or translation, and their sizes are modified by post translational modifications [28]. Furthermore, the FUS-DDIT3 protein has been reported to influence alternative splicing of some transcripts and the normal FUS is involved in translational control [29]–[31]. Reverse transcription PCR and partial exon border sequence analysis of *TP53* transcripts from MLS cell lines showed however, normal sized cDNA fragments, indicating that normal *TP53* transcripts (similar to ENST00000269305) were produced (Figure 1C). Furthermore, mass spectrometry analysis of immune precipitated TP53 protein from MLS 402-91 showed the expected peptides of the normal protein (Figures 1C and S1). The N- and C-terminal parts were not represented among the analysed fragments due to few trypsin sites in these protein regions. Antibodies directed against N-terminal, central and C-terminal epitopes reacted however, with the MLS derived proteins showing that all major parts were present. In summary, MLS cell lines expressed normal TP53 proteins detected as bands at 53 and 56 kD. The differences in TP53 size and antibody reactivity observed between MLS and HT1080 cells can best be explained by post translational modifications resulting in the 56 kD protein, which was most prominent in the HT1080 cell line (Figure 1B and 1D). Post translational modifications such as phosphorylation and methylation may lead to masking of N- and C-terminal epitopes and this can explain the low reactivity of the 56 kD band with the N- and C- terminal antibodies (Figure 1B) [28], [32], [33].

### Irradiation induced post translational modification of TP53 in MLS cell lines

Activation of TP53 by irradiation induced DNA-damage is known to involve protein modifications such as phosphorylation, methylation, ubiquitinylation and sumoylation [28], [32], [33]. These modifications lead to TP53 activation as a transcription factor and expression of downstream genes, including *CDKN1A*/*P21* [34], [35]. Western blot analysis of TP53 with the core reactive antibody revealed a 69 kD band that was highly upregulated in irradiated MLS 402-91 and 1765-92, and slightly upregulated in DL 221 (Figure 1D). This band represented a substantial proportion of the total TP53 amount and the size shift is in agreement with reported irradiation induced post-translational modification of TP53 [33]. The MLS 2645-94 cells expressed the same 69 kD band, but with no or small detected regulation. Immunofluorescence analysis of MLS cell lines 402-91, 1765-92 and 2645-94 showed expression of TP53 in almost all of the cells with weaker staining in MLS 2645-94 (Figure 2A). Consequently, only minor changes in the number of TP53 expressing cells were detected after irradiation. Detailed analysis showed that most irradiated MLS 402-91 and 1765-92 cells were strongly stained for TP53 compared to control cells, while MLS 2645-94, HT1080 and HT1080 FUS-DDIT3 cells showed few strongly TP53 stained cells in both control and irradiated cells (Figure 2A). We conclude that MLS cell lines 402-91 and 1765-92 responded as expected for normal cells with accumulation of modified TP53 protein after irradiation, whereas DL-221 and MLS 2645-94 cells responded weakly (Figures 1D and 2A). DL 221 cells express mutated TP53, but our sequence analysis failed to detect dysfunctional TP53 mutations in MLS 2645-94. The latter cell line differ, however, from MLS 402-91 and 1765-92 by the expression of a shorter FUS-DDIT3 fusion protein (type II) and by the fact that it was established by infecting the primary tumor cells with SV40 virus [5]. Thus, MLS 2645-94 is capable of expressing the full range of SV40 T antigens whereas 402-91 and 1765-92 cells express only the large T antigen [3].

The two cell lines with highest FUS-DDIT3 expression (MLS 402-91 and MLS 1765-92) showed strongest TP53 activation (Figures 1D and 2C). To test whether the FUS-DDIT3 fusion protein could interfere with TP53 expression and function, we analysed HT1080 cells with and without stably transfected FUS-DDIT3 type II protein (HT1080 FUS-DDIT3). The immunofluorescence analysis showed weak TP53 expression but substantial increase of positive cells in HT1080 FUS-DDIT3 cells (p<0.01, Figure 2A). This increase may be explained by stress effects of the oncoprotein in the transfected cells and also mirrors the high background expression of TP53 in the MLS cell lines. Irradiation of parental HT1080 cells induced increased number of TP53 positive cells (p<0.05). Most HT1080 FUS-DDIT3 cells expressed TP53 before irradiation, consequently small effects were observed after irradiation. However, irradiation increased accumulation of post-translationally modified TP53 protein in both wild type and HT1080 FUS-DDIT3 cells (Figure 1D). These data show that the irradiation induced TP53 modification was not compromised by the FUS-DDIT3 fusion protein. Consequently, the weak TP53 response observed in MLS 2645-94 can therefore most likely be explained by the SV40 infection of this cell line [25], [26].

### Irradiation induced P21 expression in MLS cells

Expression of P21 (also known as CDKN1A, WAF1 or CIP1) is induced by activated and functional TP53 after irradiation damage [28], [32]. Expression of P21 was elevated in all irradiated MLS cell lines although the induction was weaker in DL 221 cells and MLS 2645-94 (Figures 1D and 2B). HT1080 cells showed high background expression of P21 and irradiation increased P21 expression further (Figure 1D). No major difference between the parental and the FUS-DDIT3 expressing HT1080 cells was observed, showing that the fusion protein caused no inhibitory effect on TP53 induced P21 expression. Again, the failure of MLS 2645-94 to respond can be explained by the SV40 infection of this cell line.

We conclude that three commonly used SV40 T antigen or SV40 virus transformed long term passaged MLS cell lines are genetically stable on the molecular level and that they produce normal TP53 proteins. Irradiation induced normal TP53 modification and P21 expression in two SV40 T antigen transformed MLS cell lines, while one SV40 virus infected cell line and one cell line with a knownTP53 mutation failed to respond. The FUS-DDIT3 fusion protein showed no effect on irradiation induced modifications of TP53 or TP53 induced P21 expression. The infrequent mutations and normal TP53 function in MLS/RCLS explains the genetic stability of this tumor entity. In the few cases where *TP53* mutations occur, this is associated with progressive disease [15]–[17].

## Supporting Information

Figure S1 **Amino acid sequence of TP53 in MLS 402-91.** Mass spectrometry detected amino acids detected from immune precipitated materials are shown in shaded gray. Lack of representation from N- and C-terminal ends may be explained by the absence of trypsin sites in these parts of TP53. Alternating black and blue amino acids indicate exons.
(PDF)

Table S1 **Complete list of sequence variations in MLS cell lines.**
(XLSX)
