# Supplementary material for: Normal and Functional TP53 in Genetically Stable Myxoid/Round Cell Liposarcoma
Source: PLoS One. 2014 Nov 13;9(11):e113110. doi: 10.1371/journal.pone.0113110 (PMC4231113; doi:10.1371/journal.pone.0113110)
Supplement: Figure S1 — Amino acid sequence of TP53 in MLS 402-91. Mass spectrometry detected amino acids detected from immune precipitated materials are shown in shaded gray. Lack of representation from N- and C-terminal ends may be explained by the absence of trypsin sites in these parts of TP53. Alternating black and blue amino acids indicate exons. (PDF) [file pone.0113110.s001.pdf]

Supplementary Information Figure 1

MEEPQSDPSVEPPLSQETFSDLWKL**LPENNVL**SPLPSQAMDDLMLSPDDIEQWFTEDPGPDEAPRMPEAAPPVAPAPAA
PTPAAPAPAPSWPLSSSVPSQKTYQGSYGFRLGFLHSGTAKSVTCT**YSPALNKMFCQLAKTCPVQLWVDSTPPPGTRVR**
AMAIYKQSQHMTEVVRRCPHHERCSDSDGLAPPQHLIRVEGNLRVEYLDDRNTFRHSVVVPYEPPE**VGSDCTTIHYNYM**
CNSSCMGGMNRRPILTIITLEDSSGNLLGRNSFEVRVCACPGRDRRTEEENLRKKGEPHHELPPGSTKRA**LPNNTSSSP**
QPKKKPLDGEYFTLQIRGRERFEMFRELNEALELKDAQAGKEPGGSRAHSS**HLKSKKGQSTSRHKKLMFKTEGPDSD**

Figure S1. Amino acid sequence of TP53 in MLS 402-91. Mass spectrometry detected amino acids detected from immune precipitated materials are shown in shaded gray. Lack of representation from N- and C-terminal ends may be explained by the absence of trypsin sites in these parts of TP53. Alternating black and blue amino acids indicate exons.
